# Supplementary material for: Structural Behavior of Amphiphilic Triblock Copolymer P104/Water System
Source: Polymers (Basel). 2023 May 31;15(11):2551. doi: 10.3390/polym15112551 (PMC10255531; doi:10.3390/polym15112551)
Supplement: Supplementary file 1 [file polymers-15-02551-s001.zip › polymers-2422259-supplementary.pdf]

# Supplementary Materials: Structural Behavior of Amphiphilic Triblock Copolymer P104/Water System

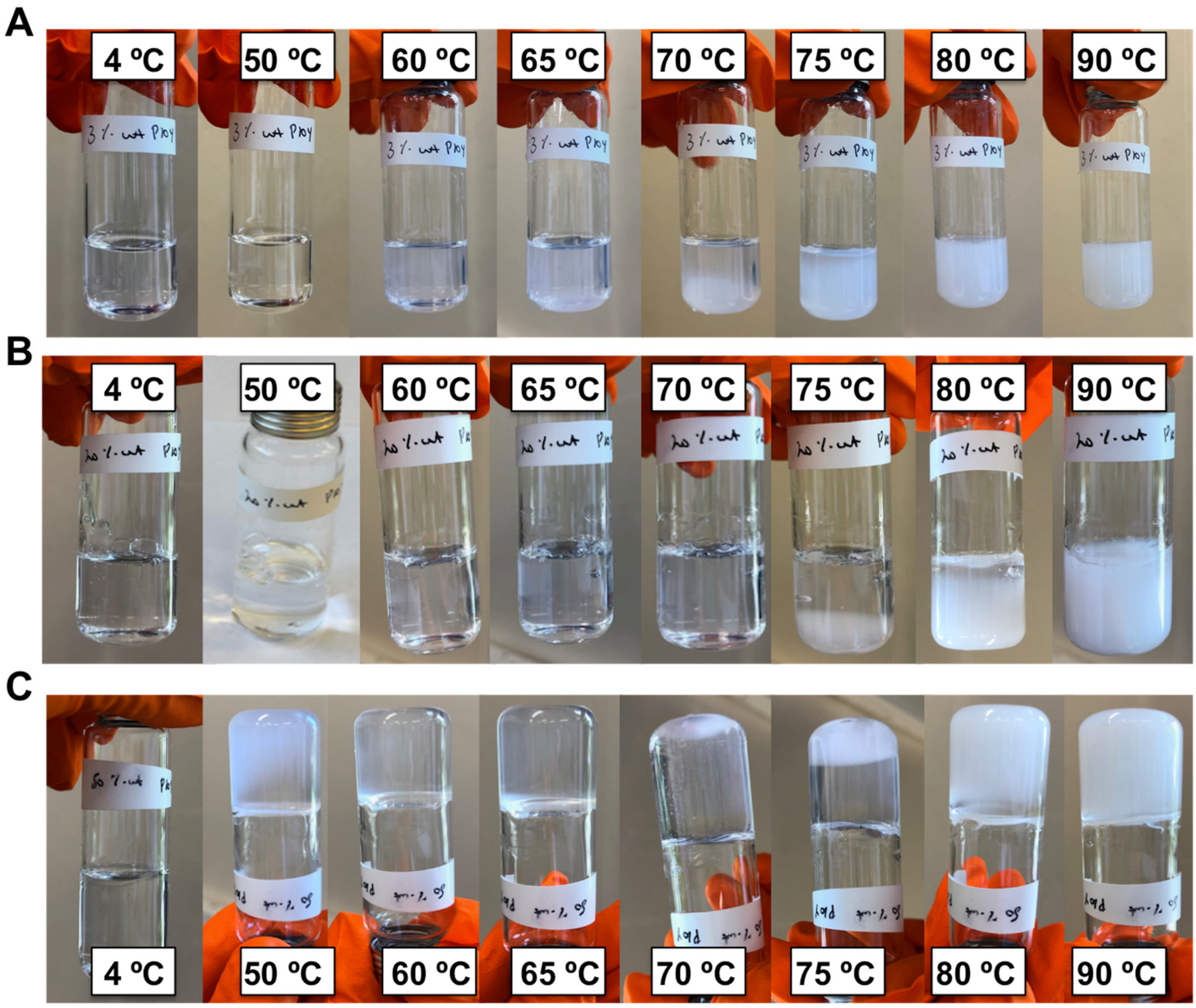


**Figure S1.** Photographs of P104/water samples having the following concentrations: A) 3, B) 20 and C) 50 wt %, taken at different temperatures (from 4 to 90 °C).

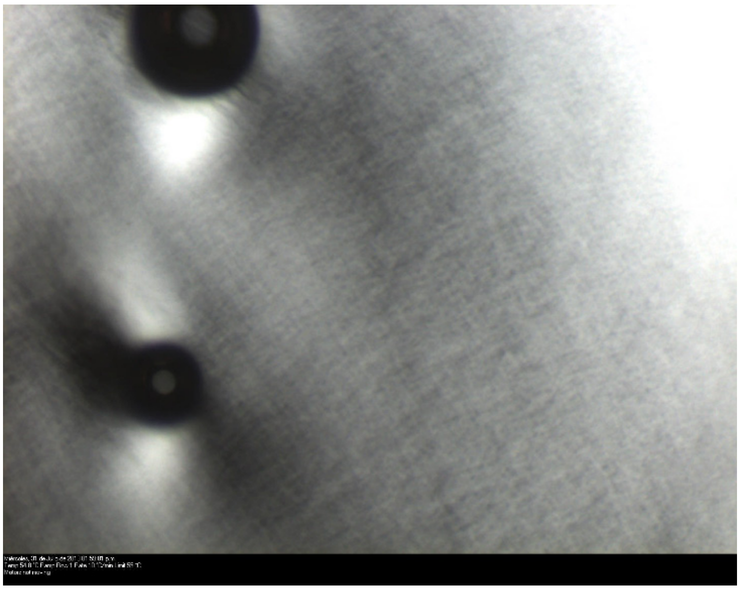

**Figure S2.** Optical micrographs obtained between crossed polarizers of P104/water solution with a concentration of 50 wt.% at 80 °C.

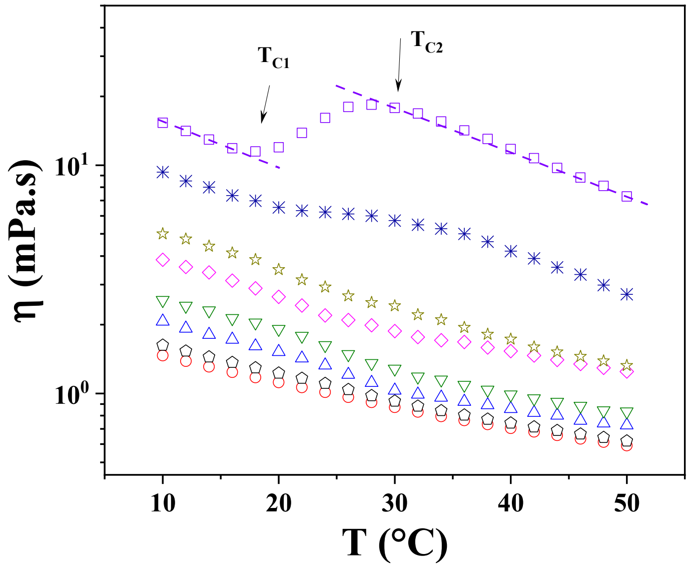


**Figure S3.** Dynamic viscosity of P104 solutions as a function of the temperature at the following $C_{P104}$: 1 (○), 2 (⬠), 3 (△), 5 (▽), 8 (◇), 10 (☆), 15 (✳) and 20 (□) wt.% Measurements performed at three different angles 30°, 50° and 70°.

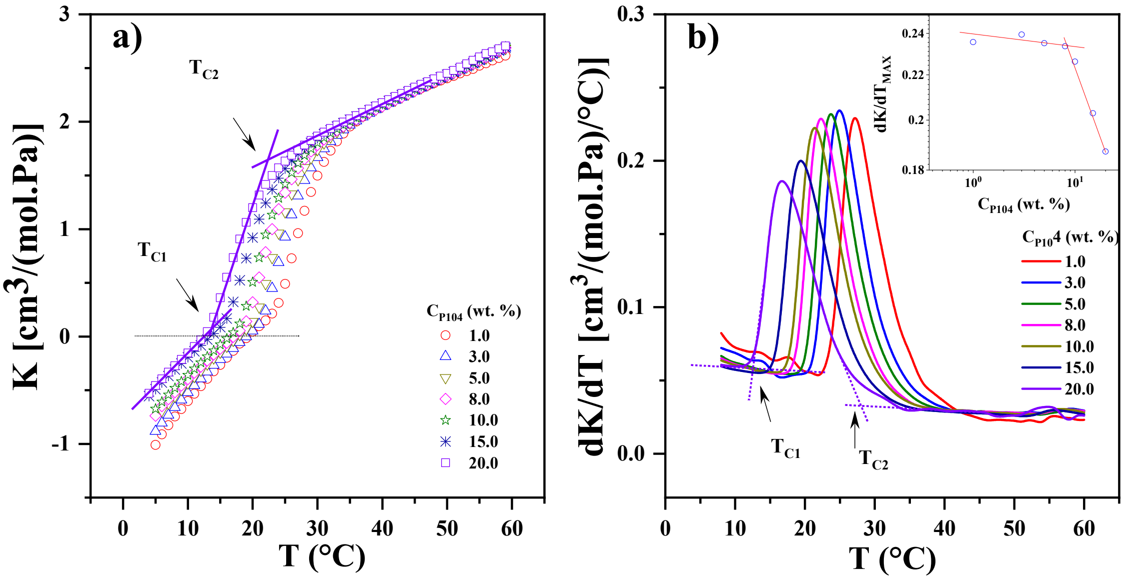


**Figure S4.** a) Apparent molar adiabatic compressibility ($K_\phi$) as a function of temperature for several P104 concentrations: 1, 3, 5, 8, 10, 15 and 20 wt%. b) Temperature dependence of apparent molar adiabatic compressibility derivative ($dK_\phi/dT$) for different P104 concentrations: 1, 3, 5, 8, 10, 15 and 20 wt%.

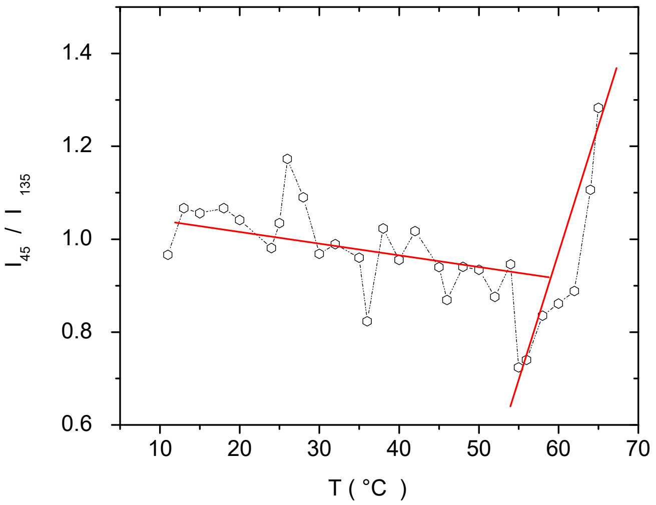


**Figure S5.** Intrinsic asymmetry [Z]=$I_{45°}$ /$I_{135°}$ as a function of temperature P104 concentration of 1 wt.%. $I_{45°}$ and $I_{135°}$ are the scattering intensity measured at 45 ° and 135 ° respectively.

a) b)

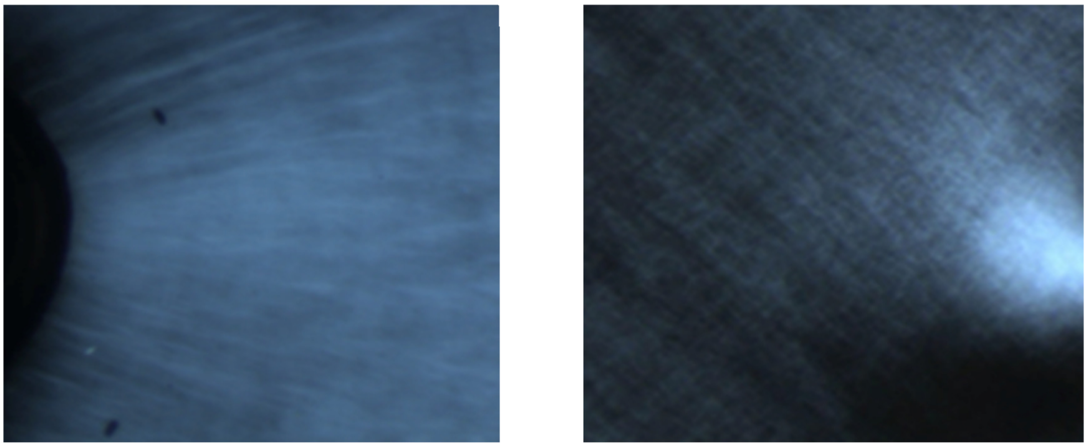

**Figure S6.** Optical micrographs obtained between crossed polarizers of P104/water solutions with the following concentrations: a) 50 wt.% at 80 °C and b) 30 wt.% at 74 °C.
